# The Yeast GSK-3 Homologue Mck1 Is a Key Controller of Quiescence Entry and Chronological Lifespan

**DOI:** 10.1371/journal.pgen.1005282

**Published:** 2015-06-23

**Authors:** Zhenzhen Quan, Lu Cao, Yingzhi Tang, Yanchun Yan, Stephen G. Oliver, Nianshu Zhang

**Affiliations:** 1 Cambridge Systems Biology Centre and Department of Biochemistry, University of Cambridge, Cambridge, United Kingdom; 2 Graduate school of Chinese Academy of Agricultural Sciences, Zhongguancun, Beijing, PR China; University of Southern California, UNITED STATES

## Abstract

Upon starvation for glucose or any other core nutrient, yeast cells exit from the mitotic cell cycle and acquire a set of G_0_-specific characteristics to ensure long-term survival. It is not well understood whether or how cell cycle progression is coordinated with the acquisition of different G_0_-related features during the transition to stationary phase (SP). Here, we identify the yeast GSK-3 homologue Mck1 as a key regulator of G_0_ entry and reveal that Mck1 acts in parallel to Rim15 to activate starvation-induced gene expression, the acquisition of stress resistance, the accumulation of storage carbohydrates, the ability of early SP cells to exit from quiescence, and their chronological lifespan. FACS and microscopy imaging analyses indicate that Mck1 promotes mother-daughter cell separation and together with Rim15, modulates cell size. This indicates that the two kinases coordinate the transition-phase cell cycle, cell size and the acquisition of different G_0_-specific features. Epistasis experiments place *MCK1*, like *RIM15*, downstream of *RAS2* in antagonising cell growth and activating stress resistance and glycogen accumulation. Remarkably, in the *ras2∆* cells, deletion of *MCK1* and *RIM15* together, compared to removal of either of them alone, compromises respiratory growth and enhances heat tolerance and glycogen accumulation. Our data indicate that the nutrient sensor Ras2 may prevent the acquisition of G_0_-specific features via at least two pathways. One involves the negative regulation of the effectors of G_0_ entry such as Mck1 and Rim15, while the other likely to involve its functions in promoting respiratory growth, a phenotype also contributed by Mck1 and Rim15.

## Author Summary

The vast majority of eukaryotic cells exist in a non-proliferating state known as G_0_. However, how cells transit into, and survive during, the G_0_ state is poorly understood. Dysregulation of the G_0_ state leads to age-related diseases such as Alzheimer’s or cancers. We have revealed that the yeast Mck1 and Rim15 kinases, which function downstream of the PKA and/or TOR signaling pathways, coordinate cell cycle progression, cell size homeostasis, and the acquisition of a variety of G_0_-specific characteristics during the transition into stationary phase. Failure of this coordination compromises the ability of early stationary-phase cells to exit from quiescence and severely shortens their chronological lifespan. Further genetic analyses suggest that the nutrient sensor Ras2 may antagonize G_0_ entry via at least two pathways, one through the negative regulation of the G_0_-specific effectors (Mck1 and Rim15) and the other possibly involving its functions in promoting respiratory growth, a phenotype also intricately modulated by Mck1 and Rim15. As Ras2 and Rim15 have homolog in both insects and/or mammals, the identification of the GSK-3 homologue Mck1 and the characterisation of its relationship with Rim15 and Ras2 in G_0_ entry could provide important clues to the regulation of these processes in more complex organisms.

## Introduction

Research into the biology of aging in different model organisms has identified several signaling pathways affecting lifespan. Among them, the partially conserved insulin/IGF-1 signaling pathway and the conserved TOR pathway regulate lifespan in organisms from insects to mammals [1–2]. Multiple TORC1-regulated processes, including autophagy, stress resistance, and mitochondrial function, contribute to lifespan extension by TORC1 inhibition [2–3]. In budding yeast, transition into quiescence and extension of chronological lifespan (CLS, defined as the period of time that non-dividing cells remain viable in the stationary phase, SP), is regulated by the TOR and PKA signaling pathway [4–5]. Compromising TOR [6–7] or deletion of the Sch9 kinase [8], a downstream effector of TORC1 [9], leads to CLS extension. Similarly, inactivation of Ras2, which promotes Cyr1 and PKA function, extends yeast life span [10]. CLS extension by reduced TOR/Sch9 signaling or decreased PKA activity is dependent on the activation of the stress response, which is mediated by the PAS kinase Rim15 and its downstream effectors, Msn2/Msn4 (Msn2/4) and Gis1 [11]. Recently, Shadel and colleagues have revealed that enhanced mitochondrial respiration above a certain threshold is required to promote cell survival during SP [12]. Increased respiration in *tor1∆* cells contributes to CLS extension through reactive oxygen species, which act as an hormetic signal to activate the stress response dependent on Msn2/4 and Gis1, and promote sub-telomeric chromatin silencing via the DNA damage response pathway [13–14]. These studies support the view that, besides other factors, the stress response induced via the inhibition of the nutrient signaling pathways is a major process involved in the prolongation of CLS [15].

The stress response mediated by Msn2/4 and Gis1, activated in cells starved for glucose or treated with rapamycin, is dependent on Rim15 [16]. The Rim15 kinase, via the paralogous Igo1 and Igo2 proteins, protects newly expressed mRNAs from decapping and degradation [17–18] and also preserves Gis1 in a phosphorylated (active) state by inhibiting PP2A^Cdc55^ phosphatase activity [19]. Our recent studies indicated that regulation of the starvation-induced stress response involves a more complex signaling network than previously thought. Firstly, Msn2/4- and Gis1-activated gene expression is negatively modulated by both the proteasome and the TOR signaling pathways [20–21]. Like Msn2 [22], Gis1 is subjected to partial cleavage mediated by the proteasome [20,23]. Secondly, when the function of the proteasome is inhibited, Msn2/4- and Gis1-dependent gene expression induced by TORC1 inhibition is no longer strictly dependent on Rim15 [24]. Yak1 (the yeast homolog of mammalian DYRKs) was identified as the kinase, acting in pathways parallel or compensatory to that of Rim15, that activates gene expression dependent on Msn2/4 and Gis1 [21]. Moreover, deletion of both *RIM15* and *YAK1* did not abolish such expression, suggesting that other genes may act to promote the stress response in TORC1-inhibited or starved yeast cells. Our examination of this hypothesis has led to the finding that the yeast GSK-3 homologue Mck1 acts in parallel to Rim15 to control the acquisition of a variety of quiescence-related characteristics; these include starvation-induced gene expression, stress resistance, accumulation of storage carbohydrates and chronological life span. Mck1 promotes cell separation and, together with Rim15, controls the cell size after the diauxic shift. Further genetic analyses suggest the nutrient sensor Ras2 may prevent G_0_ entry via at least two pathways, one through the negative regulation of G_0_-related effectors, such as Mck1 and Rim15, and the other likely involving its functions in promoting respiratory growth, a phenotype also modulated by Mck1 and Rim15. To our knowledge, this is the first demonstration that transition-phase cell cycle, cell size, and the acquisition of different G_0_-specific features are co-ordinately regulated in order to ensure long-term survival. Our findings provide novel insight into how G_0_ entry is controlled by the nutrient sensors and their downstream effectors.

## Results

### Mck1 is necessary to promote starvation-induced gene expression

**Fig 1:**
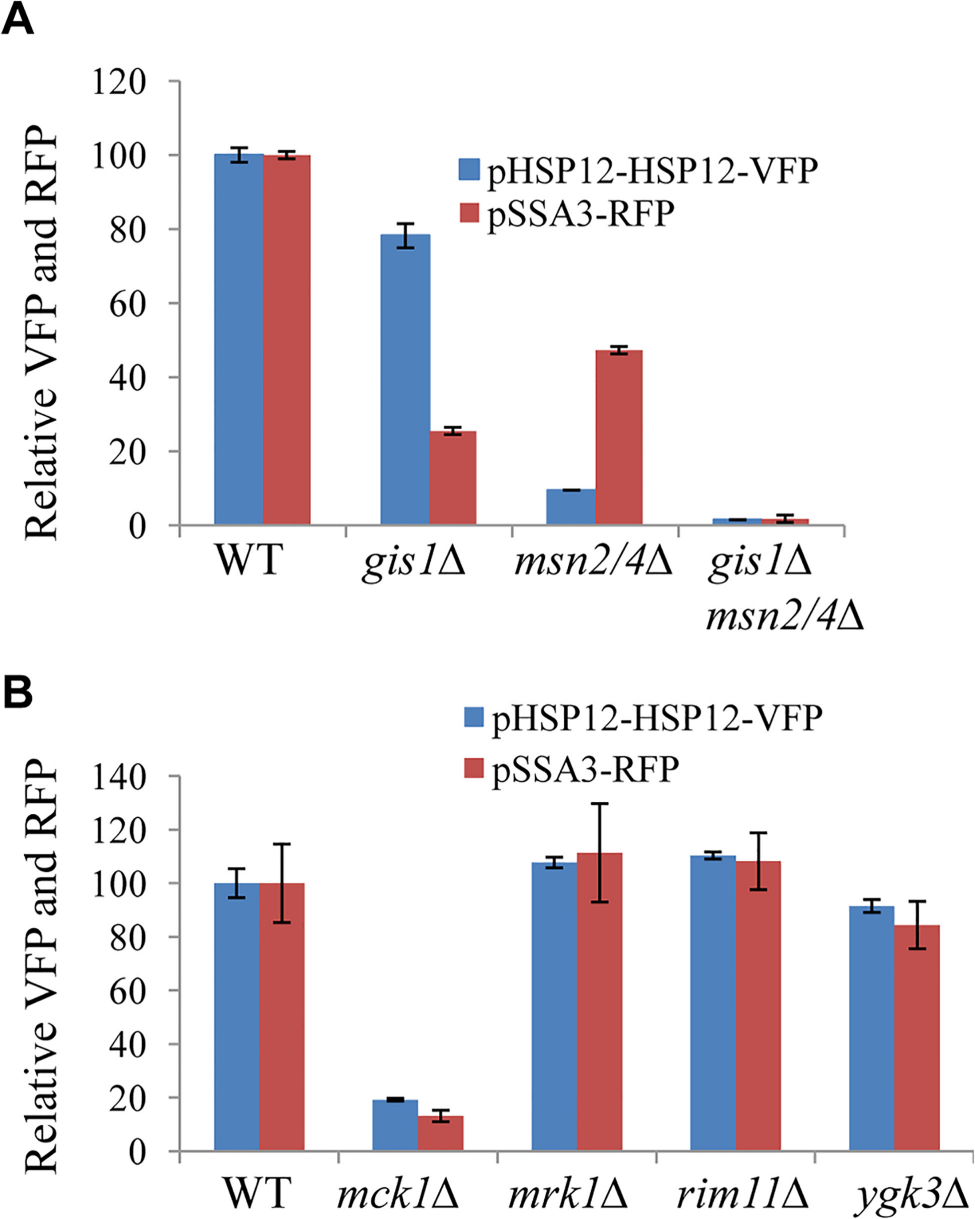
Relative expression levels of pHSP12-HSP12-VFP and pSSA3-RFP in WT, *msn2/4∆*, *gis1∆* and *msn2/4∆gis1∆* cells (A) and in mutants of GSK-3 family (B).

To facilitate the identification of other regulators of starvation-induced gene expression, two cassettes were constructed in which the expression of RFP (Red Fluorescent Protein) is regulated by the promoter of *SSA3* (harbouring the PDS motif targeted by Gis1; [25]) and that of the fusion protein HSP12-VFP (Venus Fluorescent Protein) controlled by the promoter of *HSP12* (bearing the STRE element targeted by Msn2/4, [26]). To verify the utility of the two reporters, their expression levels were first monitored in wild-type (WT), *msn2/4∆*, *gis1∆*, and *gis1∆msn2/4∆* cells using a plate reader. pHSP12-HSP12-VFP expression was evident in the late exponential phase and reached a maximum before early stationary phase (S1 Fig). In contrast, the expression of pSSA3-RFP was activated at the late exponential phase and gradually increased during the transition into stationary phase (S1 Fig). Maximum Expression of pHSP12-HSP12-VFP (at ~20h) was significantly reduced in the *msn2/4∆* cells and only moderately decreased in the *gis1∆* deletant (Fig 1A). In comparison, pSSA3-RFP expression (at ~48h) was substantially reduced in *gis1∆* cells and, to a lesser degree, in the *msn2/4∆* mutants (Fig 1A). In the *gis1∆msn2/4∆* triple mutant, the expression of pHSP12-HSP12-VFP and that of pSSA3-RFP was nearly abolished (Fig 1A). These data indicated that the two expression cassettes are suitable for monitoring starvation-induced gene expression mediated by Msn2/4 and Gis1.

Expression from the pSSA3-RFP and pHSP12-HSP12-VFP reporters was assayed following transformation into a mini-library of 272 mutants each carrying the deletion of a non-essential gene encoding a signaling molecule in *S*. *cerevisiae* (S1 Table). Mck1 was identified as being required to activate the expression of both reporters using a plate reader (Fig 1B). *MCK1* encodes a dual-specificity protein kinase, related to mammalian glycogen synthase kinases in the GSK-3 family, which has previously been shown to activate gene expression mediated by Msn2 in *S*. *cerevisiae* [27]. Among the four GSK-3 family kinases encoded by the yeast genome, only deletion of *MCK1* significantly reduced the expression of both the VFP and RFP reporters, whereas deletion of *YGK3*, *RIM11* or *MRK1* had little effect (Fig 1B).

### Mck1 and Rim15 activate starvation-induced gene expression, stress resistance, and accumulation of storage carbohydrates via parallel pathways

**Fig 2:**
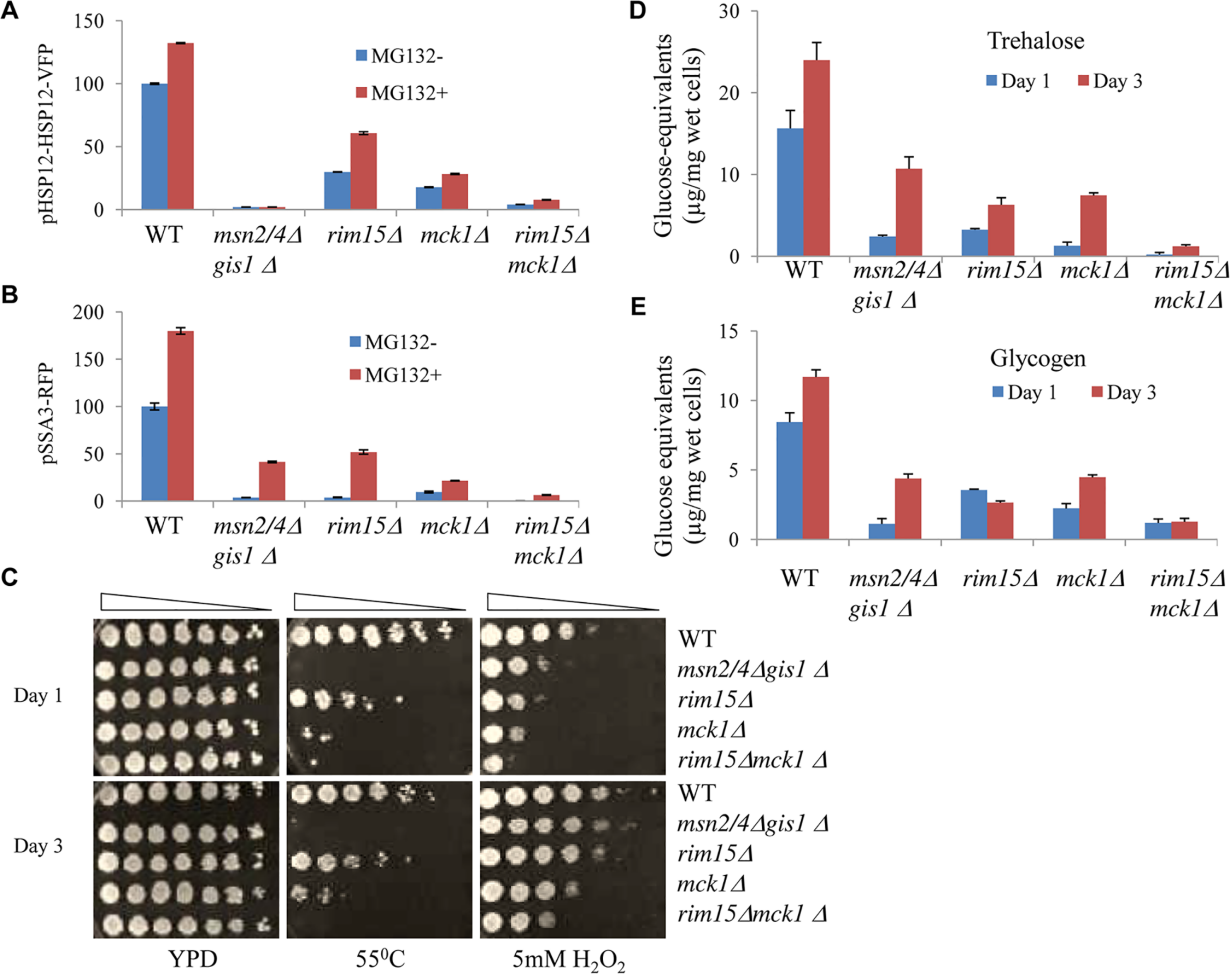
Starvation-induced gene expression, stress resistance, and storage carbohydrates detected in transition-phase cells. Relative levels of pHSP12-HSP12-VFP **(A)** and pSSA3-RFP **(B)** in WT, *msn2/4Δgis1Δ*, *rim15Δ*, *mck1Δ* and *rim15Δmck1Δ* cells treated with drug vehicle (blue, MG132-) or 12.5μM of MG132 (red, MG132+). **C**: Heat and oxidative stress resistance in transition-phase cells grown in YPD for 1 day (top) or 3 days (bottom). **D** and **E:** The amount of trehalose (**D**) and glycogen (**E)** accumulated in WT and mutant cells grown in YPD for 1 day (blue) or 3 days (red).

The Rim15 kinase was previously shown to orchestrate G_0_ entry [16]. To reveal the functions of Mck1 and its relationship with Rim15 in the regulation of G_0_ entry, single and double deletions of *RIM15* and *MCK1* were constructed in the *pdr5∆* deletion background. *PDR5* was deleted in each of the wild-type and mutant strains in order to sensitise cells to the proteasome inhibitor MG132 [28]. The levels of pHSP12-HSP12-VFP and pSSA3-RFP were monitored in cells treated with either the drug vehicle (MG132^-^) or MG132 (MG132^+^). In comparison to that seen in the single kinase mutants, the level of pHSP12-HSP12-VFP was decreased dramatically (Fig 2A) and that of pSSA3-RFP was completely abolished in the *rim15∆mck1∆* double mutants (Fig 2B). Compromising the function of the proteasome with MG132 enhanced the expression of both reporters in the WT cells (red bars in Fig 2A and 2B). Deletion of *MSN2/4* and *GIS1* abolished the expression of pHSP12-HSP12-VFP but not that of pSSA3-RFP, indicating that other factors sensitive to proteasome function may be involved in regulating *SSA3* expression. As compared to the single kinase deletants, MG132-induced expression of the two reporters was significantly reduced in the *rim15∆mck1∆* mutants (red bars, Fig 2A and 2B). These data suggest that Mck1 acts in parallel to Rim15 to activate Msn2/4- and Gis1-dpenendent gene expression.

To further examine the physiological implications of the above findings, the stress resistance conferred by cells at the transition phases (grown in YPD for 1 and 3 days) was monitored. The *gis1∆msn2/4∆* triple mutant cells were highly sensitive to heat shock (both day 1 and day 3 cultures, Fig 2C) and only moderately sensitive to oxidative stress during early transition phase (day 1 culture, Fig 2C). The *mck1∆* mutants displayed more severe defects than the *rim15∆* cells in both heat tolerance and oxidative stress resistance (Fig 2C). The *rim15∆mck1∆* double mutant recapitulates the heat shock sensitivity of the *gis1∆msn2/4∆* triple mutant and exhibited greater sensitivity to oxidative stress than the latter (Fig 2C). These data further suggest that the two kinases may control the acquisition of stress resistance in parallel pathways during transition into stationary phase.

Storage carbohydrates are accumulated in yeast cells during the transition to stationary phase [29]. We determined the level of trehalose and glycogen in cells grown in YPD for 1 and 3 days. Deletion of both *MSN2/4* and *GIS1* led to a substantial decrease of trehalose and glycogen levels in transition-phase cells (Fig 2D and 2E), indicating that the accumulation of storage carbohydrates is in part the subject of transcriptional control. In the *rim15∆mck1∆* mutants, the amount of trehalose or glycogen was lower than that in the single kinase mutants (Fig 2D and 2E), suggesting that Mck1 and Rim15 act in parallel to control the levels of both storage carbohydrates. The levels of trehalose and glycogen in the *rim15∆mck1∆* mutants were significantly lower than those seen in the *msn2/4∆gis1∆* triple mutants at 3 days of growth. The above data suggest that Mck1 and Rim15 have additional roles in determining the accumulation of storage carbohydrates other than by controlling the transcription mediated by Msn2/4 and Gis1.

**Fig 3:**
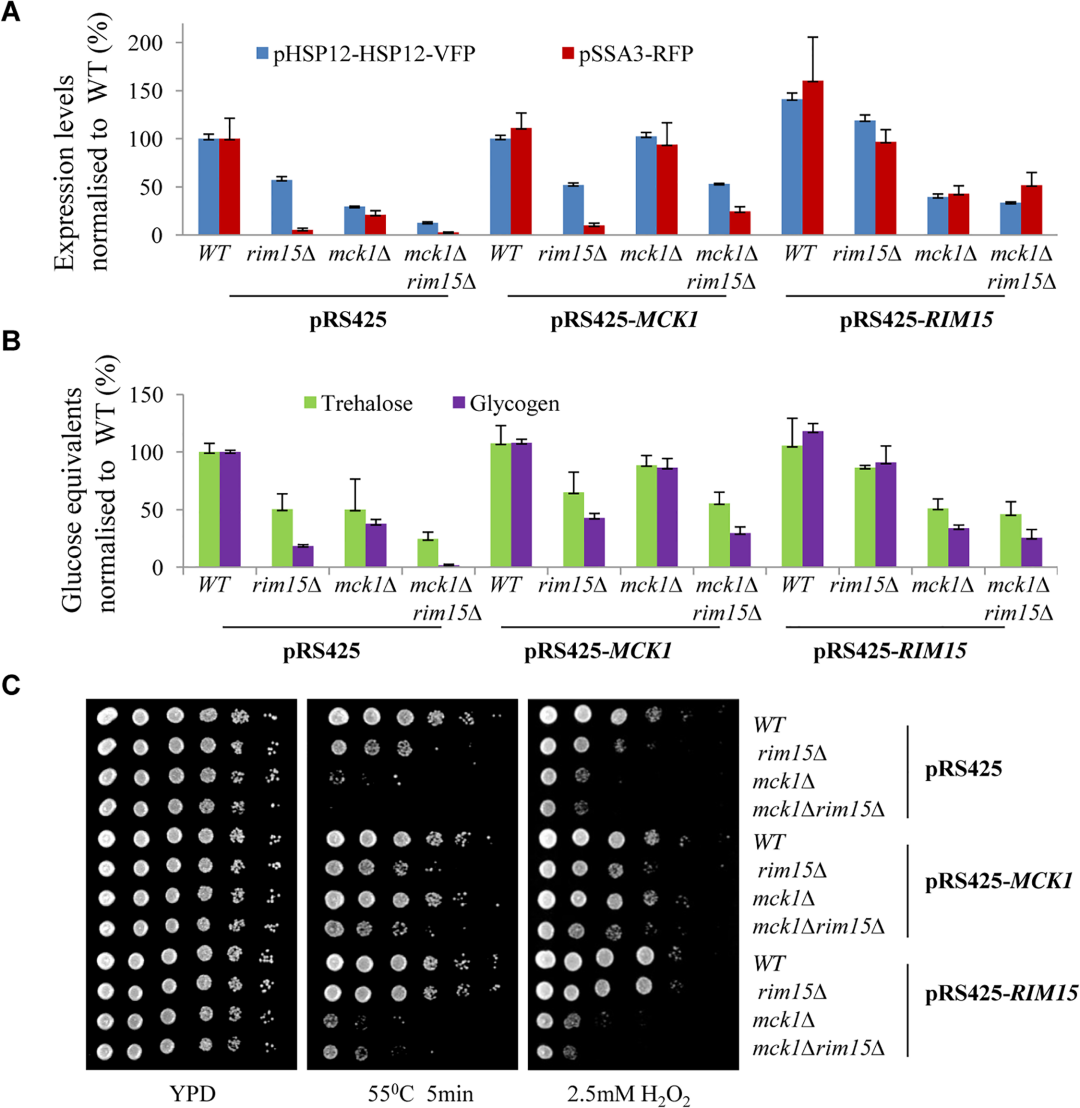
Relative expression levels of pHSP12-HSP12-VFP and pSSA3-RFP (A), relative levels of storage carbohydrates (B) and stress resistance (C) displayed by WT, *rim15∆*, *mck1∆* and *rim15∆mck1∆* cells overexpressing *MCK1* or *RIM15*.

*MCK1* and *RIM15* under the control of their endogenous promoters were also over-expressed in WT, *rim15∆*, *mck1∆* and *rim15∆mck1∆* cells using a multi-copy plasmid. Overexpression of *MCK1* marginally increased the expression levels of the two reporters in WT cells, as compared to those seen in the same cells harbouring the empty vector (Fig 3A). In contrast, *RIM15* overexpression seemed to significantly increase the levels of both reporters in WT cells (Fig 3A). Overexpression of *MCK1* restored the expression levels of the two reporters in the *mck1∆* mutants to that seen in WT cells but failed to rescue the reporter expression defects in the *rim15∆* cells or in the *rim15∆mck1∆* double mutants (Fig 3A). Similarly, *RIM15* in a multi-copy plasmid largely suppressed the gene expression defects observed in the *rim15∆* mutants but not those seen the *mck1∆* deletants. *RIM15* overexpression in the *rim15∆* mutants or *MCK1* overexpression in the *mck1∆* cells did not fully restore the level of pSSA3-RFP to that seen in the WT cells, possibly due to the decreased plasmid stability in post-diauxic shift cells. These observations confirmed that Mck1 and Rim15 operated in parallel pathways to activate gene expression dependent on Msn2/4 and Gis1.

Trehalose and glycogen levels were also determined in WT and mutant cells harbouring the multi-copy plasmids. Cells were grown in buffered SC medium [30] for 2 days. As shown in Fig 3B, *MCK1* and *RIM15* overexpression restored trehalose and glycogen in the *mck1∆* and *rim15∆* mutants respectively close to their WT levels. Overexpression of *RIM15* did not significantly increase the levels of trehalose and glycogen in the *mck1∆* mutants (Fig 3B). *MCK1* overexpression, however, partially suppressed the defects of storage carbohydrate accumulation seen in the *rim15∆* cells (Fig 3B). The above cell cultures were grown on YPD medium containing H_2_O_2_ or subjected to heat shock before growing on YPD. *RIM15* overexpression completely rescued the defects of heat or oxidative stress resistance seen in the *rim15∆* cells but not those observed in the *mck1∆* mutants (Fig 3C). *MCK1* overexpression completely restored the stress resistance capabilities of the *mck1∆* deletion cells and seemed to weakly suppress the stress resistance defects of the *rim15∆* cells (Fig 3C). The above observations further supported that *RIM15* and *MCK1* act largely in parallel to promote the transition from exponential growth to stationary phase. However, it cannot be excluded that the two kinases may interact to regulate the accumulation of storage carbohydrates under certain conditions (see later results).

### Mck1 and Rim15 regulate cell cycle progression and cell size during transition into SP

**Fig 4:**
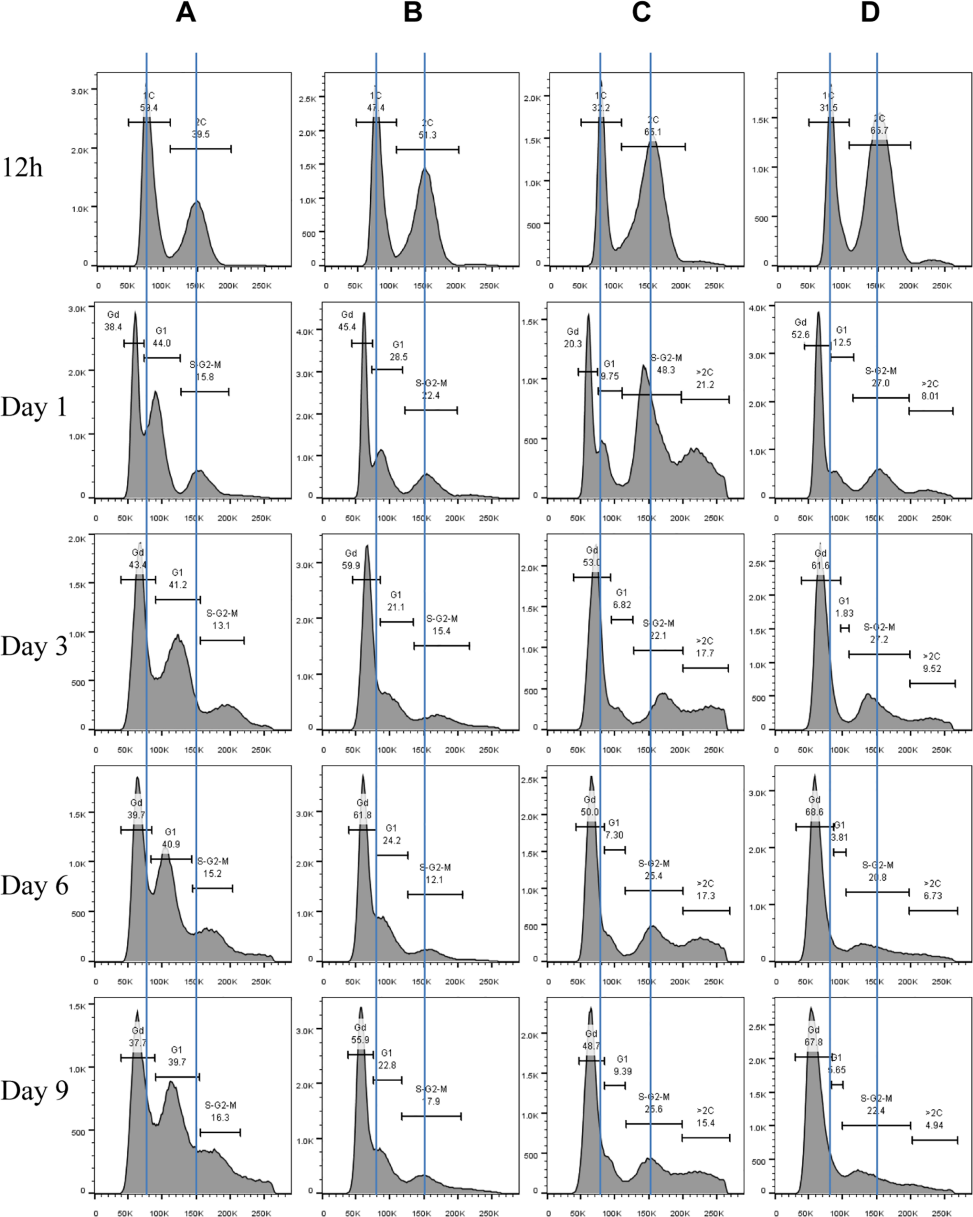
Histograms of Sytox green staining signals in WT (A), *rim15∆* (B), *mck1∆* (C), and *rim15∆mck1∆* (D) cells at the diauxic shift (12h), day 1, day 3, day 6 and day 9. A minimum 10, 000 cells were analysed for each sample.

**Fig 5:**
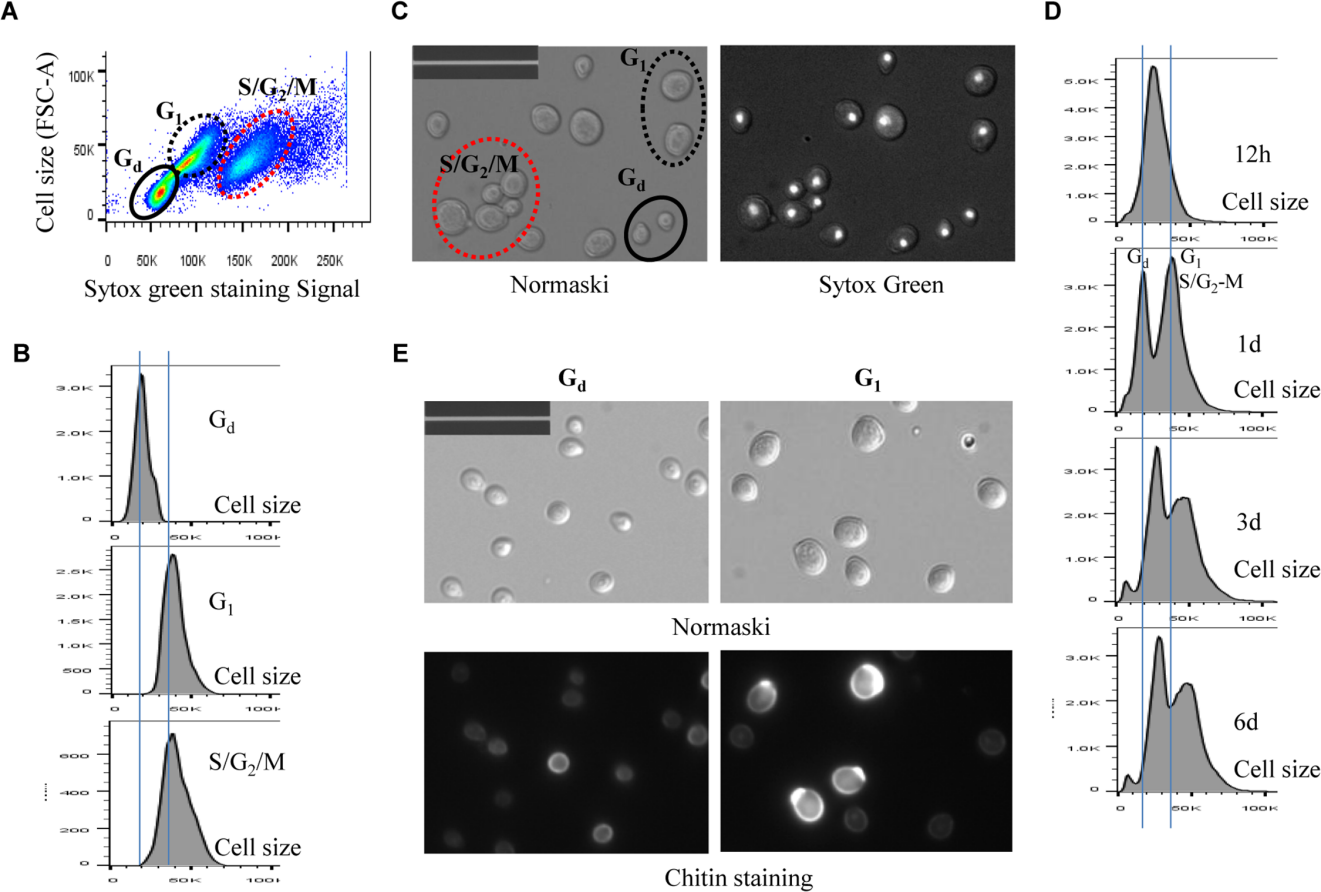
Analysis of cell size of different populations in WT cell culture. 5A: Scatter plot showing Sytox green staining intensity and the size (FSC-A) of WT cells grown in YPD for 1 day. **5B**: Histogram showing the size distribution of G_d_, G_1_ and S/G_2_/M populations in WT cell culture grown for 1 day. **5C:** Nomarski and Sytox green staining micrographs of WT cells grown for 1 day. Bar: 20μm. **5D:** Histograms showing the size distribution of WT cells grown for 12 hours (12h), 1 day, 3 days and 6 days. **5E:** Sorted G_d_ and G_1_ cells stained by calcofluor white. Bar: 20μm.

Exit from the mitotic cell cycle is one of the characteristics associated with entry into quiescence [4]. We wished to find out whether the Mck1 and Rim15 kinases also play a role in controlling cell cycle progression during the transition to stationary phase (defined as >7 days in YPD after diauxic shift). Hence the above WT, single- and double-mutant cells grown over the period of 9 days were fixed, sonicated and subjected to FACS and budding index analyses. Glucose was totally consumed by WT and mutant cells after 12 hours of growth in YPD. At 12 hours, the WT culture consisted of two distinct populations, labelled as 1C and 2C in Fig 4A. During the post-diauxic shift phase, three distinct populations, labelled G_d_, G_1_ and S/G_2_/M in Fig 4A, were accumulated in wild-type cell cultures. The G_d_ cells display less staining by Sytox green than 1C cells at diauxic shift. G_1_ cells exhibit slightly stronger staining signals than 1C cells, whereas S/G_2_/M cells have similar DNA fluorescence to that of 2C cells (Fig 4A). G_d_ cells are significantly smaller than G_1_ or S/G_2_/M cells, as revealed by the forward scatter (FSC) of the cytometer (Fig 5A and 5B). In contrast, G_1_ and S/G_2_/M cells have a similar average size and similar size distributions (Fig 5A and 5B).

**Fig 6:**
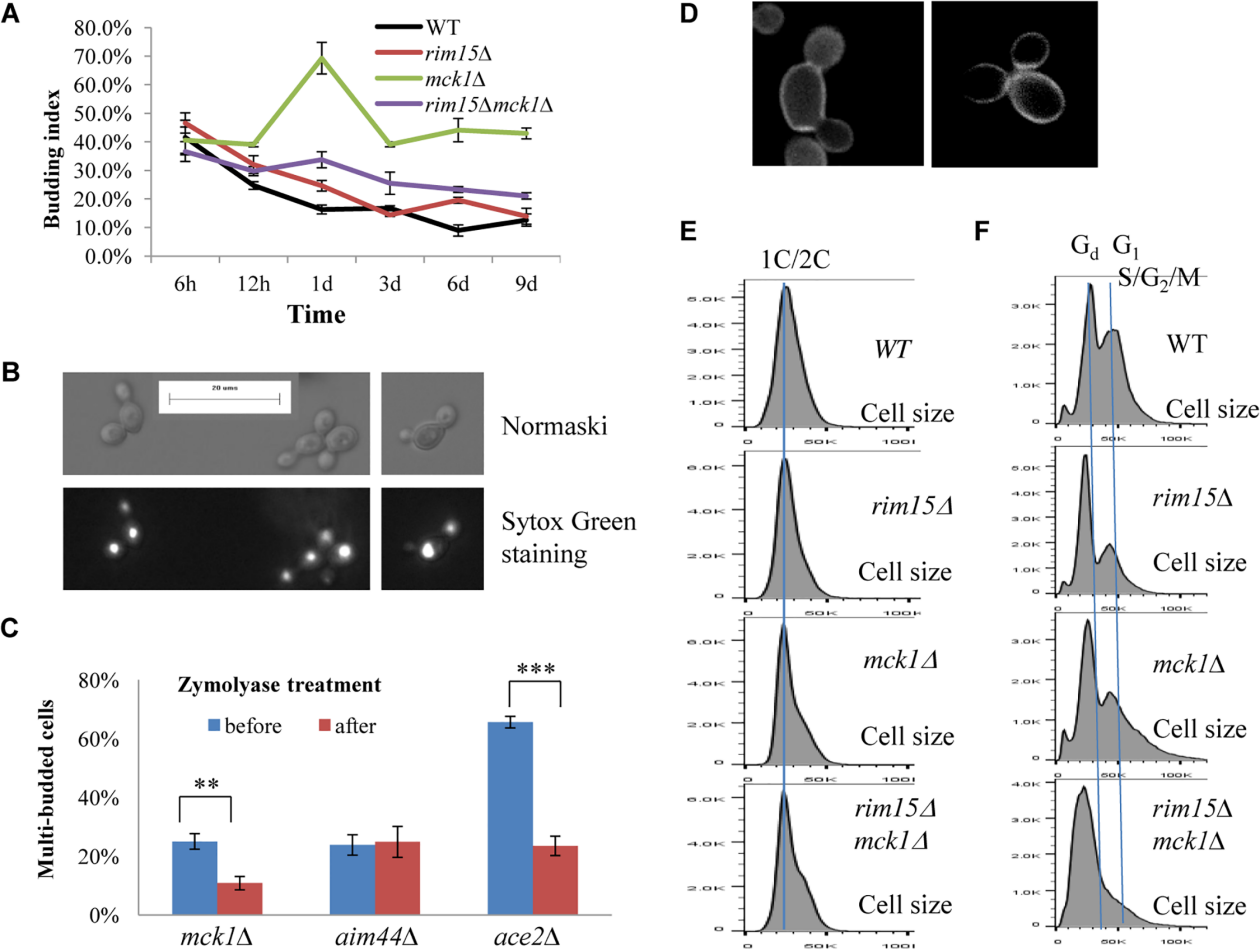
Cell size and cell separation defects displayed by the *mck1∆* mutants. 6A: Budding index during the transition into stationary phase. **6B**: Nomarski and Sytox green staining micrographs of *mck1∆* cells grown in YPD for 1 day. **6C:** Percentage of multibudded *mck1Δ* cells before and after zymolyase treatment. ** means 0.001<p<0.005, and *** indicates p<0.001. **6D:** Multibudded *mck1Δ* cells showing glucan staining at one bud neck (left) or both bud necks (right)**. 6E**: Histograms showing the size distribution of WT, *rim15∆*, *mck1∆* and *rim15∆mck1∆* cells grown in YPD for 12 hours. **6F**: Histograms showing the size distribution of WT, *rim15∆*, *mck1∆* and *rim15∆mck1∆* cells grown in YPD for 3 days.

Imaging the cells (day 1 sample) also revealed three distinct cell types: small cells without buds, large cells without buds, and large cells with a very small bud or with nuclear DNA-containing buds (Fig 5C), representing G_d_, G_1_ and S/G_2_/M populations respectively (Fig 5A and 5B). Average Sytox staining signals produced by G_d_ cells marginally increased during the transition to SP (Fig 4A). In contrast, staining signals generated by G_1_ cells increased dramatically until day 3 and then decreased slightly at the late stages of the transition (compare 12h, day 1, day 3 and day 6 samples in Fig 4A). A similar increase and decrease was seen for S/G_2_/M cells after day 1 (Fig 4A). The average size of G_d_, G_1_ and S/G_2_/M cells was similarly increased during early transition (compare day 1 and day 3 samples in Fig 5D) and remained unchanged during late transition (compare day 3 and day 6 samples in Fig 5D). The budding index was decreased from **~**40% at the diauxic shift (12h) to **~**10% at day 6 (Fig 6A). Similar FACS analyses of post-diauxic shift cell cultures from a different genetic background [31] also revealed three cell types, with the small cells displaying less DNA fluorescence than 1C, and their size increasing during the transition into SP. Although it is not clear what caused the difference of DNA fluorescence between small G_d_ and large G_1_ cells or among the G_1_ and S/G_2_/M cells during the transition (see Discussion), the above findings do confirm that cultures in transition phase exhibit an heterogeneous and dynamic structure and arrest predominantly in the G_1_ phase of the cell cycle [31]. The G_d_ and G_1_ cells were also sorted and subjected to calcofluor white staining. None of the small G_d_ cells (~200) has a bud scar (Fig 5E). In contrast, around 30% of the G_1_ cells (~200) were found to have one or more bud scars (Fig 5E). Less than 2% of the G_1_ cells are budded. These data indicated that the G_d_ population is composed of small daughter cells, whereas the G_1_ population represents predominantly daughter cells which have grown in size and old mother cells.

FACS analysis of exponential phase culture revealed similar distributions of 1C and 2C cells in the WT, *rim15∆*, *mck1∆* and *rim15∆mck1∆* cultures (S2 Fig). At the diauxic shift, the ratio of 2C to 1C populations in the *rim15∆*, *mck1∆* or *rim15∆mck1∆* cultures was significantly higher than that seen in WT culture (12h samples in Fig 4A, 4B, 4C and 4D). During the post-diauxic phase, the ratio of G_1_ to G_d_ cells was dramatically decreased in the *rim15∆* culture as compared to that of the WT (compare day 1, day 3 and day 6 samples between Fig 4B and 4A). Enhanced Sytox staining of G_1_ or S/G_2_/M cells seen in wild-type culture was also significantly reduced in the *rim15∆* culture. The fraction of S/G_2_/M population in the *rim15∆* culture, however, was only slightly higher at day 1 and dropped to the level similar to that in the WT culture thereafter (compare all samples between Fig 4B and 4A). These data indicate that deletion of *RIM15* produces G_1_ cells which are less distinguishable from the G_d_ population by FACS than those in the WT culture. At the diauxic shift (12h), the *rim15∆* mutant cells displayed similar average sizes and size distributions to those WT cells (Fig 6E). During the post-diauxic phase, the average size of G_d_ or G_1_ and S/G_2_/M cells in the *rim15∆* culture was smaller than those in the WT culture (Fig 6F). These data suggest that Rim15 is necessary to promote cell growth to transit from G_d_ (small) to G_1_ (large) during the post-diauxic shift phase.

Strikingly, the proportion of both G_d_ and G_1_ cells in the *mck1∆* culture was severely decreased during the early transition phase (12h to day 1 in Fig 4C). In contrast, a significant portion of the *mck1∆* cells (labeled >2C) contained three times as much DNA as the G_d_ cells (day 1 sample in Fig 4C). The percentage of cells in this population decreased initially but remained at about 15% of the culture during the late transition (day 3 to day 9 in Fig 4C). Correspondingly, the budding index of *mck1∆* cells increased from ~40% at the diauxic shift to ~70% at day 1, decreasing to, and remaining at, ~40% during later transition (Fig 6A). About one third of the *mck1∆* budding cells have two buds, one or both of which have acquired nuclear DNA (Fig 6B). Multi-budded cells are hallmarks of cytokinesis or cell separation defects. To distinguish between the two possibilities, we treated the *mck1∆* cells (grown in YPD for 1 day) with zymolyase. Similarly treated were the *aim44∆* and the *ace2∆* mutant cells, which have been shown to have cytokinesis and cell separation defects respectively [32–33]. Zymolyase treatment led to a significant decrease of multi-budded cells in the *mck1∆* population, similar to that observed for the *ace2∆* culture (Fig 6C). The percentage of multi-budded *aim44∆* cells remained unchanged (Fig 6C). This suggested that the *mck1∆* mutants have defects in cell separation during the transition to SP. Around 90% of the multi-budded cells (~130) displayed glucan staining at one or both bud necks (Fig 6D). Towards the end of cytokinesis, two secondary septa are formed at both sides of the primary septum and are made of 1,3-β-D-glucans and mannoproteins [34]. Glucan staining at the bud neck further indicated that the multi-budded *mck1∆* cells were caused by defects in cell separation.

At the diauxic shift (12h), the *mck1∆* and the *rim15∆mck1∆* double mutants, like the *rim15∆* mutants, also displayed similar average sizes and size distributions to WT cells (Fig 6E). During the post-diauxic phase, the average size of G_d_ or G_1_ and S/G_2_/M cells in the *mck1∆* culture was similar to those in the *rim15∆* culture but smaller than the same cells in the WT culture (Fig 6F). Further, deletion of *RIM15* in the *mck1∆* mutant led to the complete disappearance of G_1_ cells from the FACS profiles (compare day 1 with later samples in Fig 4D) and indistinguishable G_d_ and S/G_2_/M cells in terms of size (Fig 6F), indicating that Mck1 regulates the size of transition-phase cells together with Rim15. Interestingly, deletion of *RIM15* in the *mck1∆* mutant led to a significant reduction of both S/G_2_/M and the >2C populations (from 12h to day 1, compare Fig 4D with 4C) and a dramatic reduction of budding index (Fig 6A). It is likely that the extremely small G_d_ and G_1_ cells in the *rim15∆mck1∆* culture (Figs 4D and 6F) would take much longer to grow in size and to enter into the S phase, thus reducing the S/G_2_/M and the >2C populations, and the budding index as well.

### Mck1 and Rim15 synergistically control chronological lifespan

**Fig 7:**
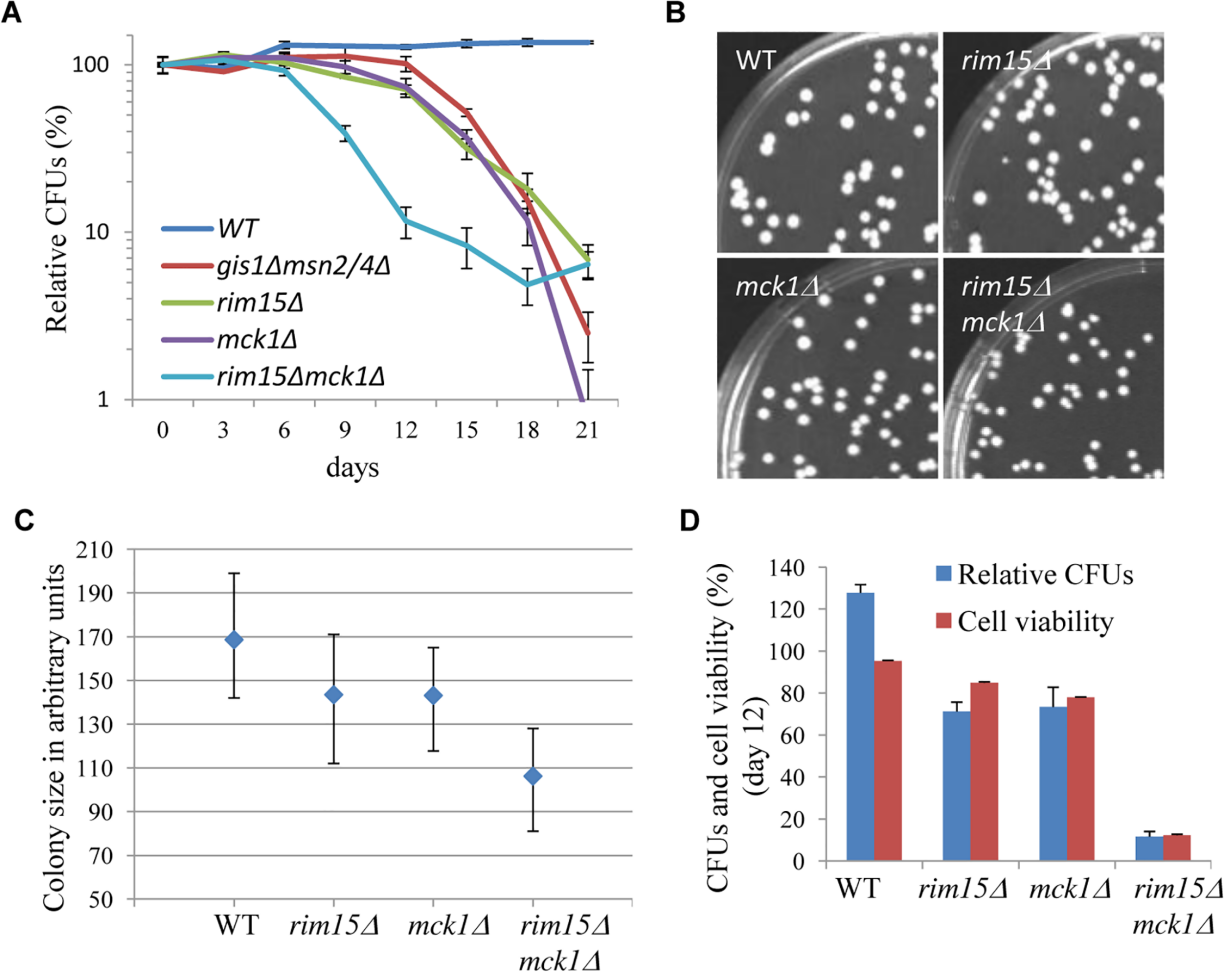
The impact of *MCK1* and/or *RIM15* deletion on quiescence exit and cell survival. 7A: Relative CFUs of WT and mutant cells taken from YPD culture every 3 days. **7B:** Cells grown from 9 day-old WT, *rim15∆*, *mck1∆* and *rim15∆mck1∆* culture for 2 days on YPD. **7C:** Quantification of colony size of cells from 6b. Lower and upper bars stand for the 1^st^ and the 3^rd^ quartiles respectively. A minimum of 200 colonies were analysed for each strain. **7D:** Relative CFUs and cell viability of stationary-phase culture at day 12.

After 3 days growth in YPD (day 0), chronological life span (CLS) was monitored by normalising the number of colony-forming units (CFUs) produced by a stationary phase culture to that produced by the preceding transition-phase culture at day 0. Due to evaporation, the culture volume tended to decrease by around 20% during the course of the study. Under the conditions of our assay, the CLS of the wild-type cells remained the same after an initial increase (Fig 7A). The initial increase may have been due to continued cell growth in YPD during the latter part of the transition phase. The *rim15∆* and *mck1∆* cells (Fig 7A) displayed a slightly lower CLS than that of the *msn2/4∆gis1∆* mutants. Deletion of both *RIM15* and *MCK1* decreased the CLS to a greater extent than removal of either kinase. The pH values of the spent media from both WT and mutant cultures fell to between 4.5 and 5, indicating that medium acidification is not a cause of the difference observed between these mutants. At day 6 (after 9 days of incubation), the majority of the stationary-phase cells can form colonies (Fig 7A). However, the size of the colonies formed by the *rim15∆* and *mck1∆* culture is smaller than that of the WT cells (Fig 7B and 7C). Deletion of both *RIM15* and *MCK1* further decreased the average size of colonies (Fig 7B and 7C). These data indicate that Mck1 and Rim15 control the ability of early SP cells to exit from quiescence. To decide whether the shortened CLS was due to defects in G_0_ exit or to cell death, cells from a culture at day 12 were washed in PBS buffer, stained with Sytox green, and cell viability determined by FACS. Deletion of *RIM15* or *MCK1* decreased the cell survival rate to ~80% (Fig 7D), while *rim15∆mck1∆* double deletants had a dramatically reduced cell viability of **~**20% (Fig 7D). These data indicate that Mck1 and Rim15 act together to regulate the proper exit from early stationary phase, and the two kinases synergistically control cell survival during prolonged starvation. Cell survival rates among the WT and the kinase mutants are highly correlated with the amount of storage carbohydrates accumulated during the transition phase (correlation coefficient: 0.69, S3 Fig) but very poorly correlated with the percentage of unbudded cells in the stationary phase culture (correlation coefficient: 0.08, S3 Fig), suggesting that signaling to accumulate sufficient storage carbohydrates rather than exit from cell cycle may be the primary determinant of the ability of SP cells to exit from G_0_ and their long-term survival.

### Mck1 may be a downstream target of the Ras/cAMP pathway

**Fig 8:**
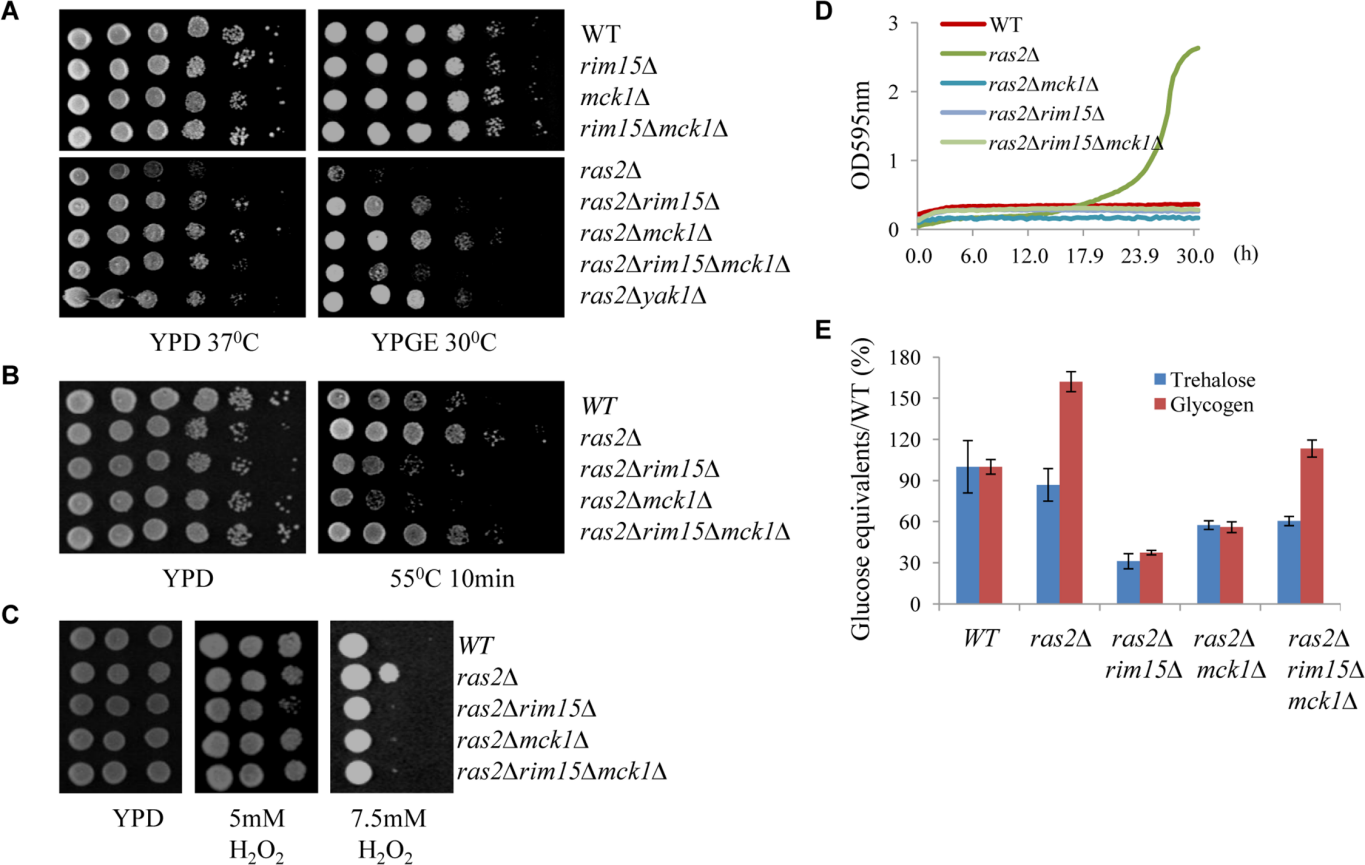
Assessing the genetic relationship between *MCK1* and *RAS2*. 8A: Suppression of the temperature sensitivity (left) and the respiratory growth defects (right) of the *ras2Δ* mutants by the deletion of *RIM15*, *MCK1* or both. **8B:** Heat tolerance displayed by the *ras2∆*, *ras2∆rim15∆*, *ras2∆mck1∆* and *ras2∆rim15∆mck1∆* cells. **8C:** Oxidative stress resistance displayed by the above cells. **8D:** Growth of the above cells in YPD liquid medium containing 0.5 mM *tert*-butyl hydroperoxide. **8E:** Trehalose and Glycogen accumulated in the above cells.

The Ras proteins sense the nutritional status of the cell and regulate the cAMP-PKA pathway by activating the adenylate cyclase Cyr1 [35]. Inactivation of *RAS2* was shown to decrease the intracellular cAMP level by four-fold [36]. We attempted to address whether *MCK1* is a downstream target of the Ras/cAMP pathway. The *ras2∆* mutant cells were shown to have growth defects at high temperatures [37–38] or on non-fermentable carbon sources [36,39]. Removal of *MCK1* largely suppressed the temperature sensitivity of the *ras2∆* cells (Fig 8A, left) and their growth defects on non-fermentable carbon sources (Fig 8A, right). Similarly, removal of *RIM15* or *YAK1* abrogated the two growth defects displayed by the *ras2∆* mutants (Fig 8A, left and right). The Rim15 kinase is negatively controlled by PKA and removal of *RIM15* has been shown to suppress the growth defect of a *cyr1* ^ts^ allele [40]. Loss of Ras activity is suppressed by the disruption of *YAK1*, which is also negatively regulated by PKA [41–42]. These data suggest that *MCK1*, could also be a growth antagonist functioning downstream of the PKA pathway (see discussion). Expression of *MCK1* under the control of the *GAL1* promoter is not toxic to cell growth of the WT or the *ras2∆* mutants (S4 Fig), indicating that the endogenous level of Mck1 is sufficient to execute its function. In this respect, we have already shown that *MCK1* overexpression on a multi-copy plasmid did not significantly increase starvation-induced gene expression in WT cells (Fig 3A). Removal of both *MCK1* and *RIM15* in the *ras2∆* cells did not further enhance cell growth at high temperature (Fig 8A, left) but rather decreased the cell growth on non-fermentable carbon sources as compared to the *ras2∆* cells in which either *MCK1* or *RIM15* is deleted (Fig 8A, right), suggesting that *MCK1* and *RIM15* are required for respiratory growth. We have previously revealed that deletion of *RIM15* rendered cells unable to compete with their WT counterparts in chemostat cultures limited for glucose or containing ethanol as the sole carbon source [20].

The *ras2∆* mutants have been shown to have increased resistance to oxidative and heat stresses [11,43]. The increased resistance to heat shock displayed by the *ras2∆* mutants (grown in YPD for 3 days) was strongly dependent on *MCK1* or *RIM15* (Fig 8B). Removal of both *MCK1* and *RIM15*, compared to deletion of either of them in the *ras2∆* cells, however, elicited increased heat stress resistance to the level similar to that of the WT cells (Fig 8B). This observation is contrary to what was seen for the *rim15∆mck1∆* cells which displayed more severe defects to heat stress than the *rim15∆* or *mck1∆* single mutants (Figs 2C and 3C). The increased heat stress resistance shown by the *ras2∆* and *ras2∆rim15∆mck1∆* cells seemed to be correlated with their compromised respiratory growth (Fig 8A, right). Reduced respiration could lead to the generation of less reactive oxygen species in yeast cells, thus increasing their capacity for stress resistance. Alternatively, other stress response pathways could be activated in the *ras2∆* cells in a way dependent on their respiratory capacity (see later results). At the median level of H_2_O_2_ (5mM), oxidative stress resistance shown by the *ras2∆* cells was strongly dependent on Rim15 but not on Mck1 (Fig 8C). The strong dependence on Rim15 was abolished when *MCK1* was further removed (Fig 8C). The *ras2∆* deletants demonstrated enhanced resistance to oxidative stress than their WT counterparts only at very high concentration of H_2_O_2_ (7.5mM, Fig 8C) or when cells were grown in liquid medium containing a more stable oxidative reagent, *tert*-butyl hydroperoxide (Fig 8D). This enhanced resistance is abolished when *MCK1* and/or *RIM15* is removed (Fig 8C and 8D), indicating that *MCK1*, like *RIM15*, acts downstream of *RAS2* to activate the oxidative stress response.

As compared to WT cells, the *ras2∆* mutants accumulated slightly less trehalose but significantly more glycogen (Fig 8E). The accumulation of both storage carbohydrates in the *ras2∆* mutants is strongly dependent on *MCK1* and, to a greater extent, on *RIM15* (Fig 8E). Further removal of *RIM15* from the *ras2∆mck1∆* cells marginally *increased* the amount of trehalose but significantly *enhanced* the accumulation of glycogen, to the level similar to that in the WT cells (Fig 8E). As revealed in Fig 2D, the glycogen level in the *rim15∆mck1∆* cells was only around 15% of that in the WT cells. These data further confirmed that in the *ras2∆* mutants, other cellular pathways are activated/deactivated to enhance the accumulation of glycogen and the acquisition of stress resistance. Glycogen synthesis in batch cultures begins before glucose is exhausted and reaches its peak before diauxic shift [29]. Glycogen stores are dropped slightly during the early adaptation to respiratory growth and then refilled thereafter to serve as energy depot during extended starvation [44]. The defective respiratory growth elicited by *RAS2* deletion may allow the build-up of glycogen stores to continue during the early transition phase. This respiratory growth defect is strongly suppressed by removal of *RIM15* or *MCK1* and the suppression is remarkably reversed by deletion of *RIM15* and *MCK1* together (Fig 8A). These data suggest that the defective respiratory growth may be one of the contributing factors to account for the excessive accumulation of glycogen in the *ras2∆* mutants and the substantial increase of glycogen levels in the *ras2∆rim15∆mck1∆* triple mutants. Taken together, our data suggest that the nutrient sensor Ras2 may prevent G_0_ entry via at least two pathways. One involves the negative regulation of the G_0_-specific effectors including Mck1 and Rim15, while the other may involve its functions in promoting respiratory growth, a phenotype also intricately regulated by Mck1 and Rim15. It will be interesting to clarify how *RAS2* promotes respiration and to understand how Mck1 and Rim15 contribute to this metabolic reprogramming.

## Discussion

**Fig 9:**
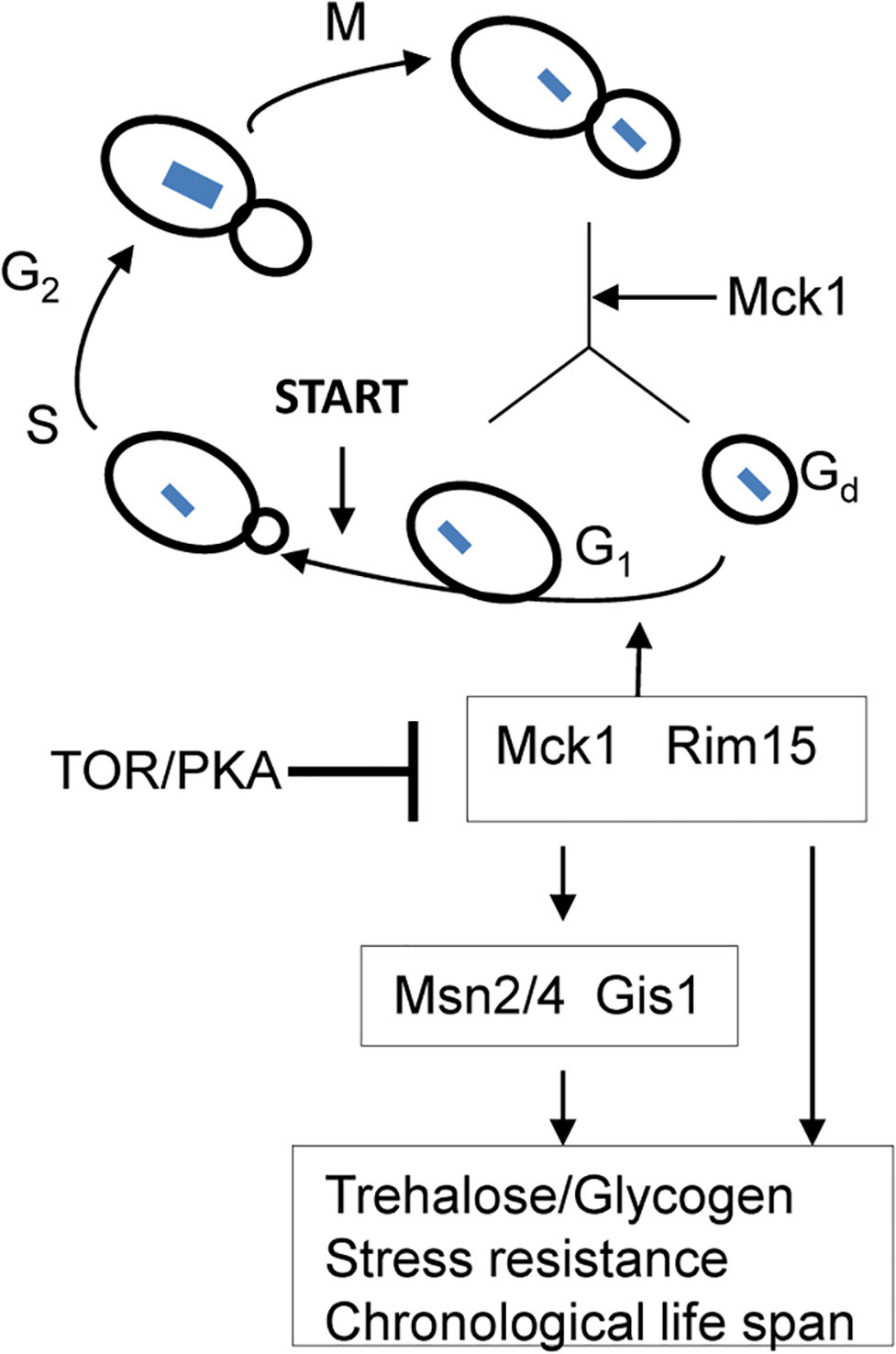
A model showing that transition-phase cell cycle, cell size and the acquisition of quiescence-related characteristics are coordinated by Mck1 and Rim15. Arrows indicate activation and bars denote inhibition. Dashed line means suggested interaction. Blue squares indicate chromosomal DNA.

There has been some debate over whether exit from the cell cycle is a prerequisite for entry into quiescence. Using density gradients, a subset of SP cells, termed Q cells, have been purified and this population is uniformly arrested at G_1_ and displays high thermotolerance and longevity [45–46], suggesting that exit from mitosis is required for the establishment of the quiescent state. Laporte *et al*. [47] have reported that yeast cells can enter quiescence from all cell cycle phases and quiescence entry and exit primarily depend on the cells’ metabolic status, indicating that quiescence establishment can be uncoupled from the cell cycle. The key to this debate is whether the quiescence ‘programme’ operates independently of the cell cycle. Here, we have revealed that a network composed of Mck1, Rim15 and their downstream transcription factors, is activated to drive both cell cycle progression and the acquisition of a variety of G_0_-features during the transition into quiescence (Fig 9). Our data indicate that quiescence entry, including cell size homeostasis and cell cycle exit, is coordinately regulated in response to nutrient starvation (Fig 9). The ability to exit from quiescence (Fig 7B and 7C) or to survive during the stationary phase (Fig 7A and 7D), however, seem to be highly correlated with the amount of storage carbohydrates rather than the cell cycle status (S3 Fig), supporting the contention that signaling to reprogram metabolic status is the primary determinant of quiescence establishment and exit [38].

Previous studies have revealed that the function of Rim15 is negatively regulated by PKA and its nuclear localisation antagonised by TORC1 [16,40,48]. Our epistasis experiments place Mck1 downstream of Ras2 to coordinate the transition into SP (Fig 8). Mck1 has been shown to phosphorylate Bcy1 to inhibit PKA activity under heat stress [49]. Mck1 was also postulated to inhibit the activity of Tpk1 but without phosphorylating it [50]. These observations suggest that Mck1 may act at the level of PKA. Overexpression of *RIM15* did not suppress the defects displayed by the *mck1∆* mutants (Fig 3) and Mck1 was shown to act largely in parallel to Rim15 to activate the expression of a variety of G_0_-related features (Fig 2). Therefore, it is unlikely that *RIM15* functions in a linear pathway downstream of *MCK1*. It is more likely that Mck1 also acts downstream of the Ras/cAMP pathway to regulate the transition into SP (Fig 9). Whether there is a feedback loop from Mck1 to inhibit PKA and how Mck1 is negatively regulated by PKA remains to be clarified. More recently, Mck1 was shown to inhibit ribosome and tRNA synthesis in glucose-starved or TORC1-inhibited cells [51]. Mck1-mediated phosphorylation of Elo2, a fatty acid elongase involved in sphingolipid biosynthesis, is also inhibited by TORC1 to regulate very long chain fatty acid synthesis [52]. Mammalian GSK-3 is a substrate of several kinases, including S6K1, a downstream target of TORC1 which phosphorylates GSK-3 to inhibit its kinase activity [53]. It is therefore possible that Mck1 is also regulated by the TOR pathway (Fig 9). Whether the Sch9 kinase, the yeast homologue of S6K1, phosphorylates Mck1 directly to inhibit its activity remains to be decided.

Deletion of *RAS2* leads to hyperaccumulation of glycogen [39,54]. A biochemical study attributed the hyperaccumulation of glycogen in the *ras2∆* mutants to the glycogen synthase activation state which rises continuously and reaches its peak before the diauxic shift, even though the glycogen phosphorylase activity is up to 40 times higher in the mutant than in the WT strain [55]. Whether the activation state of glycogen synthase in the *ras2∆* mutants is enhanced due to defective respiration remains to be decided. Respiratory defects shown by the *ras2∆* mutants are distinct from those displayed by mitochondrial respiratory mutants. In the latter mutant cells, both glycogen stores and the glycogen synthase activity are reduced [56–57]. Further kinetic and biochemical studies should provide clues as to how Ras2 interacts with Rim15 and Mck1 to regulate carbon metabolic switch and stress resistance.

We have revealed that Mck1 functions to promote cell separation during the post-diauxic shift phase (Fig 6C and 6D). The role of Mck1 in cell cycle control has been reported previously [58–59]. During the transition between G_2_/M phases, the Cdk1/Cdc28-Clb activity is abruptly raised to initiate mitosis. Decrease of this high Cdk1 activity is necessary to exit from the cell cycle at the end of mitosis. Mck1 is proposed to inhibit Cdk1-Clb activity after nuclear division to promote exit from mitosis via its interaction with, and phosphorylation of, Clb2 and Mih1 [58]. A delay in mitotic exit has been observed in exponentially growing *mck1∆* cells [58]. At the diauxic shift (compare Fig 4C and 4A, 12h), 2C cells were accumulated to a substantial level in the *mck1∆* culture as compared to those in the WT population. However, after the diauxic shift, the percentage of S/G_2_/M cells in the *mck1∆* culture was actually similar to or less than that of the same cells in the WT culture (Fig 4C and 4A). In contrast, the >2C cells with two nuclear DNA-containing buds were only accumulated after glucose exhaustion. These observations indicate that the kinases may play distinct roles in cell cycle control under different nutrient conditions. Recently, Rim15 was shown to promote timely entry into mitosis under temperature stress [60]. Homologs of Rim15, named Greatwall kinases, were demonstrated to regulate entry into mitosis in *Xenopus* egg extracts [61–62] and in *Drosophila* [63–64]. However, it remains to be decided whether the significant accumulation of 2C cells at the diauxic shift in the *rim15∆* and *mck1∆* culture (Fig 4B and 4C) is due to defects respectively in mitosis entry and exit.

Cell size homeostasis in budding yeast is controlled at the G_1_/S boundary called START, primarily by preventing cell division until a critical cell size is attained [65–66]. Under poor growth conditions [67–69] or in the post-diauxic shift phase [31], cell divisions are highly asymmetric, producing very small daughter cells (Fig 5E). We have shown that Mck1 and Rim15 modulate the size of all the cell types, especially the G_1_ and S/G_2_/M populations (Fig 6F), suggesting that the two kinases are essential for mitotic cells to grow to the required size before commitment to the next cell cycle (Fig 9). The small-size phenotype ascribed to the *mck1∆* mutants has previously been revealed by a genome-wide screen in stationary-phase cultures [70]. In glucose-limited chemostat cultures of prototrophic strains, the cell cycle is spontaneously synchronised with periodic bursts of glycolysis and respiration [71–72]. Metabolic and transcriptomic studies on these periodic cycles have revealed that storage carbohydrates are accumulated during early G_1_, which are then liquidated at late G_1_ to drive metabolism and gene expression important for growth and respiration [71–72]. Based on the link between metabolism, cell growth, and the cell cycle, Futcher [73] hypothesised that the accumulation of storage carbohydrates is an important determinant of START and cell size under nutrient-limited conditions. Furthermore, storage carbohydrates provide a ready source of energy when division resumes and are crucial to long-term survival [74]. Our findings that cell size (Fig 6F), resumption of growth from SP (Fig 7B) and chronological lifespan (Fig 7A and 7D) are closely correlated with the amount of storage carbohydrates accumulated in the WT and *rim15∆/mck1∆* mutants (Fig 2D and 2E) seem to support Futcher’s hypothesis.

Among the three distinct cell types, G_d_ cells displayed less DNA fluorescence and smaller cell size than 1C cells at the diauxic shift (Figs 4A, 6E and 6F). Small-size cells with less than 1C DNA signals have previously been reported in the post-diauxic shift cell culture [31] or in the diauxic shift culture overexpressing *CLN3* [75]. Moreover, G_1_ and S/G_2_/M cells in the *mck1∆* or *rim15∆* mutant population are smaller (Fig 6F) and they display lower DNA staining signals than those in the wild-type population (day 3 samples in Fig 4). G_1_ cells in the *mck1∆rim15∆* double mutant population have the same size distribution as G_d_ cells (Fig 6F) and these two populations cannot be differentiated by FACS (Fig 4D). These data appear to support the contention that cell size is an important factor influencing DNA staining or fluorescence detection (although for unknown reasons). However, in WT cell culture, the cell sizes of G_d_, G_1_ and S/G_2_/M cells are similarly increased from day 1 to day 3 (Fig 5D), DNA staining signals produced by G_d_ cells increased only marginally but those generated by G_1_ and S/G_2_/M cells were enhanced significantly (Fig 4A), suggesting that other mechanisms may contribute to the substantial increase of DNA fluorescence signals in large mother or mother-to-be cells. qPCR experiments confirmed that the ratio between mitochondrial and genomic DNA remained constant at ~35 from 12h to day 3 in the wild-type cell culture (S5 Fig), thus ruling out the possibility that mitochondrial biogenesis leads to increase of DNA signals in large cells. Previous studies have demonstrated that yeast cells duplicate their chromosomes (or segments of chromosomes) to overcome proteotoxic stress [76], adverse environmental conditions [77], DNA damage [78], and nutrient-limitation [79]. Similarly, *E*. *coli* cultures at stationary phase contain cells with several chromosomes and those exclusively composed of cells with a single chromosome are never observed, regardless of the growth medium [80]. Whether genomic duplications occur in starved cells and how cell size affects DNA staining or fluorescence signal detection needs to be further clarified. Future work should also aim to elucidate how Mck1 regulates cell size, cell separation and other characteristics associated with quiescent cells and how the activity of Mck1 is regulated by the PKA and TOR pathways. This knowledge, when combined with similar known interactions for Rim15 [19], should provide a mechanistic insight into both quiescence entry and the maintenance of longevity.

## Materials and Methods

### Strains and plasmids

Strains carrying single-gene deletions were obtained directly from the BY4742 mutant library (Open Biosystems). Strains carrying deletions in multiple genes were generated by combining mutations via either mating and dissection, or by PCR-mediated gene replacement using drug resistance or nutritional markers [81–82]. Deletion mutants of genes coding for non-essential signaling molecules used in this study are selected based on data in Lee *et al*. [51], and include protein, lipid and metabolite kinases, phosphatases and their regulators. Expression reporter cassettes were constructed in pRS426 in which the transcription of cds coding for red fluorescent protein (RFP, [83]) is controlled by the *SSA3* promoter. Similarly, the cds encoding Venus fluorescent protein (VFP, [84]) was fused with that of *HSP12* and expressed from the *HSP12* promoter. *RIM15* and *MCK1* under the control of their endogenous promoters were cloned into pRS425 for overexpression studies. The coding sequence of *MCK1* was also inserted into pYES2 vector downstream of the *GAL1* promoter.

### Fluorescence detection and quantification

For quantitative assays of pSSA3-RFP and pHSP12-HSP12-VFP levels in liquid cultures, freshly-grown overnight cultures were inoculated (5% v/v) into SMM medium containing 0.6% glucose which had been dispensed into 96-well microtiter plate. Cell density (OD_595nm_) and fluorescence intensities were simultaneously monitored in triplicate using a plate reader (BMG Biotech). RFP is excited at 580/±10nm and emits at 610/±10nm, while VFP is excited at 500/±10nm and emits at 540/±10nm. MG132 (a proteasome inhibitor) was added into the growing cell cultures at the early-to-mid exponential phase. The working concentration was 12.5μM for MG132 (50mM stock solution in DMSO or absolute ethanol). Medium-only blanks and WT cells bearing the same constructs were included for each run as negative and positive controls, respectively. After background subtraction, RFP and VFP fluorescence intensities were normalised to cell density. The mean and standard deviation were calculated at each time point; for simplicity, standard errors at regular intervals only are plotted with the means in all Figures.

### Determination of storage carbohydrates

The concentrations of glycogen and trehalose (μg glucose per mg of wet cells) was determined following the procedures described by Parrou and Francois [85]. Briefly, cells (**~**30mg wet weight from 1ml of culture grown in YPD) were treated with 250μl of 0.25M Na_2_CO_3_ at 95°C for 4 hours, neutralised with 0.15ml of 1M acetic acid and 0.6ml of 0.2M Na-Acetate buffer (pH = 5.2). Half of the culture was treated with trehalase (0.025u, Sigma) and incubated at 37°C overnight with shaking. The other half was treated with amyloglucosidase (0.6u, Roche) and incubated at 57°C overnight with shaking. The amount of glucose liberated into the supernatant was determined using a glucose assay kit (Sigma).

### Phenotypic assays

Stress resistance conferred by cells grown to the transition phases (1 and 3 days) was determined according to Wei *et al*. [11]. Yeast cells were subjected to treatment at 55°C for 5 or 10 minutes, serially diluted and spotted onto YPD agar to determine their resistance to heat shock. Similarly, cells were directly spotted onto YPD agar containing 2.5, 5 or 7.5mM H_2_O_2_ to assay their resistance to oxidative stress. Chronological life span (CLS) of WT and mutant cells was measured for 21 days by counting colony-forming units (CFUs) on YPD agar plates after serial dilutions. CLS was determined every three days by normalising CFUs of stationary-phase cells to that produced by a day 0 culture (after 3 days of growth in YPD). Cell viability at day 12 (day 15 in SP) was also measured by FACS analysis, essentially following the protocol described by Ocampo and Barrientos [86]. Instead of using propidium iodide, 2μM of Sytox Green was used to stain the cells. Excitation was performed using a laser at 488nm and emission detected with a standard 530/30 band pass filter.

### Analysis of cell size, cell cycle, budding index and presence of nuclear DNA in buds

SP cells were serially diluted and spread on YPD plates. Cell colonies grown at 30°C for 36–48 hours were imaged and colony size analysed with Cell Profiler (http://www.cellprofiler.org/) using the modified pipeline described by Vokes and Carpenter [87]. Cell cycle status was determined according to Haase and Reed [88] using Sytox Green to stain DNA in fixed cells. Samples were sonicated at low power (2 min) and analysed using a cytometer (LSRFortessa, Becton Dickinson). Data were processed using FlowJo software (www.flowjo.com). Images of cells were taken with a microscope (Olympus BX51) and the objective lenses (Plan N 10×/0.25), captured using QICAM (Q 24720) and Qcapture Pro 6 as the acquisition software. The percentage of budded cells was determined by counting from photographs of these images using ImageJ softwaere (http://imagej.nih.gov/ij/). To decide whether nuclear DNA was present in buds, fluorescent images of cells stained with Sytox green were captured using UPlanSApo (60×/1.35) as the object lenses. These images were exported and processed in Adobe Photoshop CS4.

### Miscellaneous

The G_1_ and G_d_ cells were sorted from the WT culture (treated and stained with Sytox Green) using a Biorad S3 sorter. Sorted cells were collected and resuspended in 50mM Tris buffer (pH 7.5) and stained with 0.1mg/ml of calcofluor white for 20min at 30°C. Stained cell were washed twice with buffer before imaging. Cells subjected to glucan staining were fixed with 70% ethanol for one hour, washed with PBS buffer twice, and stained with 5mg/ml of Aniline Blue for 20min.

## Supporting Information

S1 Fig Expression levels of pHSP12-HSP12-VFP (1a) and pSSA3-RFP (1b) in WT cells grown to the stationary phase. (PDF)

S2 Fig Histograms showing Sytox green staining signals in exponentially-growing WT, *rim15∆*, *mck1∆* and *rim15∆mck1∆* cells. (PDF)

S3 Fig Correlation between survival rate and storage carbohydrates (3a) or between survival rate and percentage of unbudded cells (3b) in WT, *mck1∆*, *rim15∆* and *mck1∆rim15∆* mutants. (PDF)

S4 Fig Effects of *MCK1* overexpression on growth of WT and the *ras2∆* cells. (PDF)

S5 Fig The copy number of mitochondrial DNAs in WT cells grown in YPD for 12h, 1, 3, 6 and 9 days. (PDF)

S1 Table The list of deletion strains used in the screening. (XLSX)
