# Supplementary figures and images for: The Yeast GSK-3 Homologue Mck1 Is a Key Controller of Quiescence Entry and Chronological Lifespan
Source: PLoS Genet. 2015 Jun 23;11(6):e1005282. doi: 10.1371/journal.pgen.1005282 (PMC4477894; doi:10.1371/journal.pgen.1005282)

**Supplementary Figure 1:**

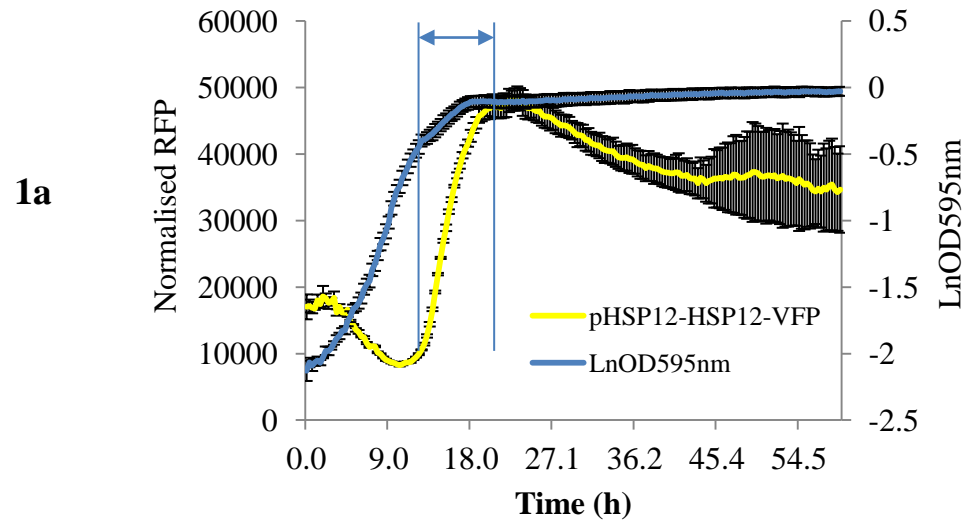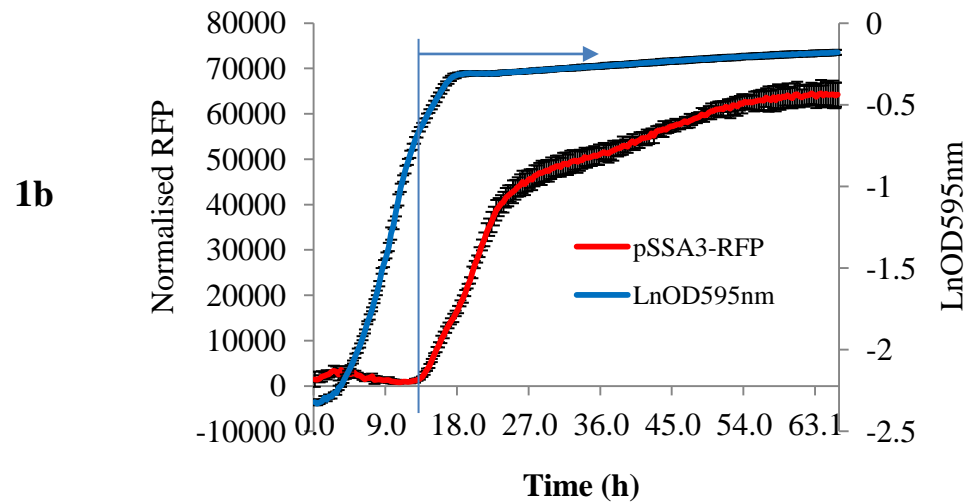

Supplement: S1 Fig — (PDF) [file pgen.1005282.s001.pdf]

## Supplementary Figure 2

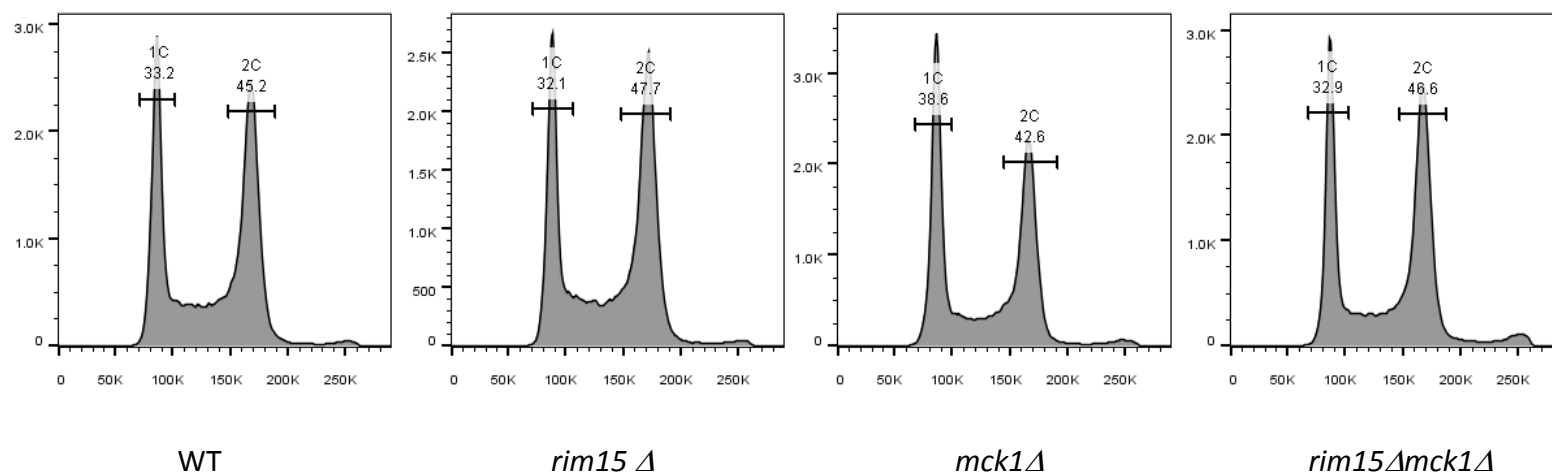

Supplement: S2 Fig — (PDF) [file pgen.1005282.s002.pdf]

### Supplementary Figure 3

3a

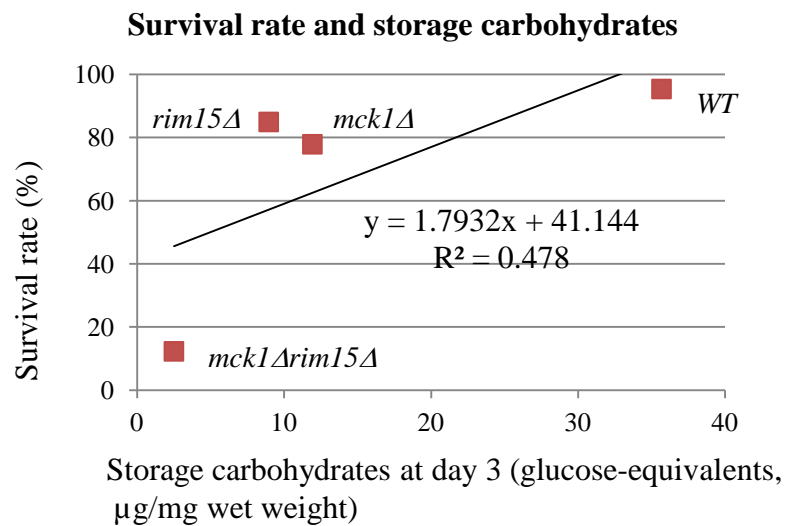

3b

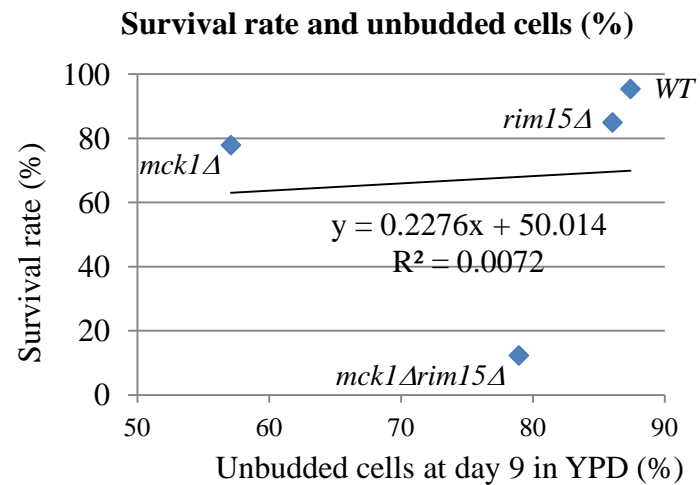

Supplement: S3 Fig — (PDF) [file pgen.1005282.s003.pdf]

### Supplementary Figure 4

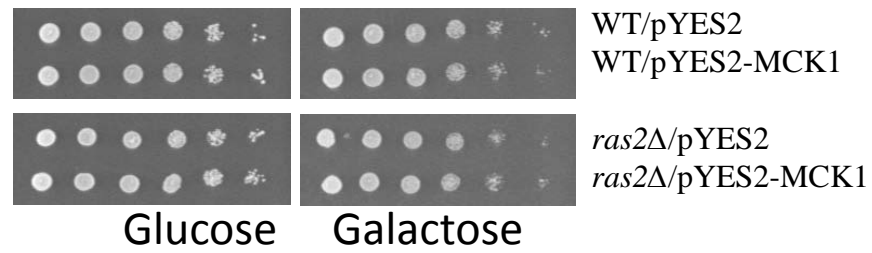

Supplement: S4 Fig — (PDF) [file pgen.1005282.s004.pdf]

**Supplementary Figure 5**

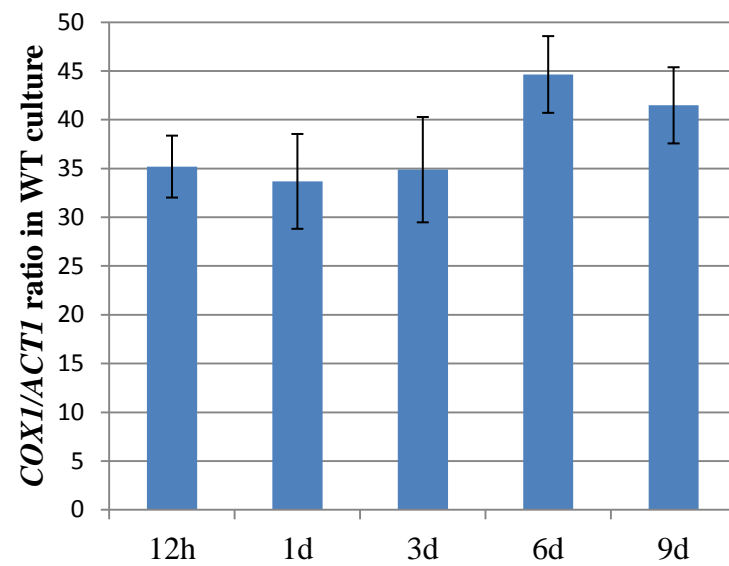

Supplement: S5 Fig — (PDF) [file pgen.1005282.s005.pdf]
